# HIV-Associated Neurocognitive Disorder (HAND) and Alzheimer’s Disease Pathogenesis: Future Directions for Diagnosis and Treatment

**DOI:** 10.3390/ijms252011170

**Published:** 2024-10-17

**Authors:** Mohammed Mustafa, Dominique Musselman, Dushyantha Jayaweera, Andrea da Fonseca Ferreira, George Marzouka, Chunming Dong

**Affiliations:** 1 Department of Medicine, Jackson Memorial Hospital, University of Miami Miller School of Medicine, Miami, FL 33136, USA; mxm194120@med.miami.edu (M.M.); djayawee@med.miami.edu (D.J.); 2 Department of Psychiatry and Behavioral Sciences, University of Miami Miller School of Medicine, Miami, FL 33136, USA; dmusselman@med.miami.edu; 3 Interdisciplinary Stem Cell Institute, University of Miami Miller School of Medicine, Miami, FL 33136, USA; axd1272@miami.edu; 4 Division of Cardiovascular Disease, Department of Medicine, Miami VA Health System, University of Miami, Miami, FL 33136, USA

**Keywords:** HIV-associated neurocognitive disorder, Alzheimer’s disease, neurocognitive impairment, biomarkers, exosomes, imaging, pathophysiology, antiretroviral therapy

## Abstract

HIV-associated neurocognitive disorder (HAND) and Alzheimer’s disease (AD) are two neurocognitive disorders with overlapping clinical presentations and pathophysiology. The two have been thought to be two separate entities. However, the introduction and widespread use of antiretroviral therapy (ART) has altered the clinical manifestations of HAND, shifting from a pattern of subcortical dementia to one more akin to cortical dementia, resembling AD. Thus, the line between the two disease entities is not clear-cut. In this review, we discuss the concept of Alzheimer’s disease-like dementia (ADLD) in HIV, which describes this phenomenon. While the mechanisms of HIV-associated ADLD remain to be elucidated, potential mechanisms include HIV-specific pathways, including epigenetic imprinting from initial viral infection, persistent and low viral load (which can only be detected by ultra-sensitive PCR), HIV-related inflammation, and putative pathways underlying traditional AD risk factors. Importantly, we have shown that HIV-specific microRNAs (miRs) encapsulated in extracellular vesicles (EV-miRs) play an important role in mediating the detrimental effects in the cardiovascular system. A useful preclinical model to study ADLD would be to expose AD mice to HIV-positive EVs to identify candidate EV-miRs that mediate the HIV-specific effects underlying ADLD. Characterization of the candidate EV-miRs may provide novel therapeutic armamentaria for ADLD.

## 1. Clinical Manifestations and Disease Progression

Human immunodeficiency virus (HIV) is a retrovirus that primarily targets the immune system, leading to acquired immunodeficiency syndrome (AIDS) when untreated [1]. Antiretroviral therapy (ART) has significantly altered the management of HIV, turning it into a chronic condition with much improved life expectancy [1]. As a result, a spectrum of neurocognitive impairment exists in people living with HIV (PLWH) [2]. Among these neurocognitive disorders, HIV-associated neurocognitive disorders (HAND) are particularly notable for their prevalence and detrimental impact on quality of life. While earlier studies estimated that approximately 30–50% of PLWH suffer from some level of HAND [2], more recent research has suggested that these estimates may be over-represented due to inconsistencies in diagnostic criteria, such as the Frascati criteria, which do not account for educational and socioeconomic differences in this population [3,4].

HAND includes a range of conditions from asymptomatic neurocognitive impairment (ANI) and mild neurocognitive disorder (MND) to the most severe form, HIV-associated dementia (HAD). HAD is characterized by a significant functional decline in cognitive abilities [5]. Alzheimer’s disease (AD) is a progressive neurodegenerative disorder that represents the most common cause of dementia in the general population, affecting 6.9 million Americans aged 65 and older [6].

The clinical presentations of HAND and AD used to be distinct. HAND typically presented with cognitive impairments in attention, speed of information processing, and executive function [7]. Conversely, AD is primarily characterized by significant memory loss, which evolves into deficits in language, executive function, and visuospatial skills [8,9]. The progression of symptoms also provided clues for differentiation. The trajectory of HAND was often tied to the management of HIV infection, with cognitive functions showing variability over time. In some cases of HAND, cognitive abilities might stabilize or even improve with effective antiretroviral therapy [2]. On the other hand, AD is marked by a gradual and relentless decline in cognitive functions, with no periods of stabilization [10]. While both conditions may exhibit symptoms such as executive dysfunction, the early and pronounced memory impairment seen in AD serves as a key distinguishing feature. However, the introduction and widespread use of ART has impacted the clinical manifestations of HAND, shifting from a pattern of subcortical dementia to one more akin to cortical dementia, resembling that observed in AD [11]. We term this as AD-like dementia (ADLD).

## 2. Prevalences

The advent of ART has changed the landscape of HAND, with a decrease in the frequency of HIV encephalitis observed at autopsy from 54% to 15% [12]. Initially, severe forms like HAD were more common, but now milder forms are more prevalent [13]. It is estimated that 30–50% of PLWH are affected by some form of HAND, even with effective viral suppression [2,14]. Among the spectrum of HAND, the prevalence of ANI, MND, and HAD are 23.5%, 12.3%, and 5.0%, respectively [15]. On the other hand, about 10% of people age 65 and older in the U.S. have AD [16]. The prevalence increases with each decade thereafter, with 32% of people 85 and older suffering from AD [17].

## 3. HAND and AD Risk Factors

Risk factors for HAND include older age, lower CD4+ T-cell counts, higher viral loads, co-infections such as Hepatitis C, substance abuse, genetic predispositions, and the virus’s capacity to cause neuroinflammation and neuronal damage [18,19,20,21]. AD is a multifactorial and polygenic chronic disease affecting mainly older people with both genetic contributions and environmental cues. Indeed, age is the greatest risk factor overall for AD development. Genetically, the presence of the apolipoprotein E (APOE) ε4 allele is the most significant risk factor for the sporadic form of the disease, increasing the risk and lowering the age of onset in a dose-dependent manner [22]. Environmental factors include hypertension, dyslipidemia, obesity, and diabetes, similar to cardiovascular disease risk factors [23]. As PLWH live longer, these environmental risk factors are also elevated, which together with HIV-specific risk factors, contribute to ADLD development.

## 4. HAND Pathogenesis

The pathogenesis of HAND is multifaceted, involving direct viral damage to neurons, chronic immune activation, and neuroinflammation [24]. It is initiated by the entry of HIV into the central nervous system (CNS). The virus employs a ‘Trojan horse’ mechanism to breach the blood–brain barrier (BBB) using infected monocytes or T-cells. Once inside the brain, it targets microglial cells— the brain’s resident macrophages—and astrocytes to a lesser extent [25]. Chronically, this leads to an inflammatory response and consequential neuroinflammation and neurotoxicity. The progression to HAD is marked by increased severity and extent of neuroinflammation, neurotoxicity, and cognitive impairment. Despite ART, low-level viral replication in the CNS continues to play a role in this process [26].

HIV infection of the central nervous system (CNS) leads to both direct and indirect neuronal injury, contributing to Alzheimer’s disease-like pathology in people living with HIV (PLWH). Viral proteins such as glycoprotein 120 (gp120), transactivator of transcription (Tat), and negative regulatory factor (Nef) cause excitotoxicity by increasing glutamate release, leading to neuronal damage. These proteins also disrupt amyloid metabolism—Tat promotes amyloid-beta (Aβ) production by upregulating beta-secretase, while Nef inhibits Aβ degradation, leading to amyloid plaque accumulation [27].

Chronic inflammation caused by HIV-activated microglia and astrocytes increases proinflammatory cytokines such as tumor necrosis factor-alpha (TNF-α) and interleukin-1 beta (IL-1β), contributing to oxidative stress and mitochondrial dysfunction [28]. This inflammatory environment promotes Aβ production and impairs clearance, contributing to amyloid plaque deposition [29]. Additionally, HIV-induced oxidative stress leads to tau hyperphosphorylation, further exacerbating neuronal dysfunction and contributing to Alzheimer’s disease-like pathology.

**Direct Viral Effects**: The understanding of HAND’s neuropathology has significantly evolved over time, shifting from initial theories that emphasized direct viral effects leading to encephalitis and neuronal loss to a more nuanced comprehension of the complex cellular and molecular mechanisms that result in functional neuronal changes. Early pathology models focused on the infection of perivascular macrophages and the formation of microglial nodules, which were thought to be central to the neuroinflammatory processes causing cognitive impairment in HIV patients [30,31]. This perspective was supported by the chronic infection of microglia and perivascular macrophages in the central nervous system (CNS), acting as a reservoir for HIV and contributing to neuronal damage from both acute and chronic infection stages [31,32,33]. The mechanism of HAND is intricately linked to the penetration of HIV into the CNS, which involves three main pathways: infecting endothelial cells through specific chemokine receptors, breaching BBB permeability by chronic infection and inflammation, and crossing the BBB via infected monocytes, perivascular macrophages, and leukocytes, which then infect resident CNS cells [34,35,36,37].

The CD4 receptor plays a pivotal role as the primary receptor for HIV infection, initiating a complex process that leads to the fusion of the viral and cellular membranes [38]. Upon binding to CD4, the HIV envelope protein undergoes conformational changes that are crucial for the virus to enter host cells. This interaction is not only fundamental for the initial stages of HIV infection but also triggers a series of events aimed at downmodulating the viral receptor, involving viral proteins such as Negative Regulatory Factor (Nef), Envelope Glycoprotein (Env), and Viral Protein U (Vpu) [39,40]. Besides CD4, which is the primary receptor for HIV-1, chemokine receptors such as CC Chemokine Receptor 3 (CCR3), C-X-C Chemokine Receptor 4 (CXCR4), and the C-type lectins Dendritic Cell-Specific Intercellular Adhesion Molecule-3-Grabbing Non-Integrin (DC-SIGN) also play pivotal roles in mediating HIV-1 entry into the central nervous system (CNS) [41,42]. A study by Mukhtar et al. (2002) [35] revealed that primary isolated human brain microvascular endothelial cells (MVECs), which constitute the major barrier of the BBB, express significant levels of these chemokine receptors but lack CD4. These MVECs may attract the virus, representing a CD4-independent pathway for HIV/SIV entry into the CNS. The presence of DC-SIGN and L-SIGN on these cells further indicates the potential for these cells to attach viral particles on their surfaces, facilitating HIV-1 entry or infection.

**Dendritic cells**: Dendritic cells (DCs), which bridge the innate and acquired immune systems, exploit complex pathways for microbial antigen uptake and transport, allowing HIV to utilize these pathways for initial entry and dissemination [43,44]. Within dendritic cells (DCs), HIV can escape endolysosomal degradation and the antigen presentation pathway, allowing for transfer to CD4+ lymphocytes during the activation of T-cell-mediated immunity [45]. The C-type lectin receptors (CLRs), such as langerin on Langerhans cells and DC-SIGN on dermal DC subsets, can bind HIV gp120 through its mannose saccharides [46]. This interaction enhances HIV fusion with the target cell membrane via CD4/chemokine receptors or mediates entry into the endolysosomal pathway, underscoring the multi-faceted nature of HIV transmission and entry strategies that could contribute to the pathogenesis of HAND [42,46].

Recent research has highlighted the roles of various proteins and receptors in HAND neuropathology. The expression of the Insulin-like Growth Factor 2 Receptor (IGF2R) in microglial nodules and its role in HIV replication and chemokine expression have been identified as significant factors in HAND pathogenesis [30]. The Nerve Growth Factor IB-like nuclear receptor Nurr1 (NR4A2) has been shown to modulate cycles of proviral reactivation and transcriptional shutdown in microglial cells. It influences the switch between active viral replication and latency. Dysregulation of this process can contribute to the persistent viral reservoir and chronic neuroinflammation seen in HAND. Given this, NR4A2 has been proposed as a potential therapeutic target. Theories suggest that targeting NR4A2 could reduce the cycles of proviral reactivation, thereby limiting viral replication and associated neurotoxic effects. Additionally, the modulated activity may attenuate the neuroinflammatory responses that contribute to HAND [47].

**Exosomes**: Exosomes are extracellular vesicles (EVs) responsible for cell-to-cell communication, signal transduction, and cellular transport. Preliminary studies have highlighted the intricate roles of exosomes in the pathogenesis of HAND, suggesting their potential as both biomarkers for and mediators of HIV-induced neuroinflammation. The HIV-1 regulatory protein Nef has been shown to enhance the release of Nef-containing exosomes from astrocytes and microglia, which can disrupt neuronal function and the integrity of the blood-brain barrier, contributing to neuroinflammation and potentially exacerbate HAND [48,49,50]. Similarly, the HIV trans-activating regulatory protein (Tat) has been implicated in altering exosomal content, including miRs, leading to decreased neuronal viability and increased neurotoxicity, further underscoring the role of exosomes in HIV-related neurotoxicity [51,52,53,54,55].

**Epigenetics**: Epigenetic regulation is crucial in gene expression and cellular activities, including the brain’s response to HIV-1 infection. MicroRNAs (miRs), small non-coding RNAs that modulate gene expression, have been implicated in controlling neuroinflammation and microglial activation [56]. Specifically, miR-124, which is highly expressed in the brain and critical for maintaining microglial quiescence, has been shown to be dysregulated by HIV-1 Tat [57]. This study demonstrated that simian immunodeficiency virus (SIV) infection in rhesus macaques and exposure of microglial cells to HIV-1 Tat protein resulted in the downregulation of miR-124 and increased DNA methylation, suggesting a complex interplay between HIV-1 Tat, miR-124 dysregulation, and epigenetic modifications in the pathogenesis of HAND. Furthermore, downregulation of miR-124 by HIV-1 Tat involves DNA methylation of miR-124 promoters and affects the MECP2-STAT3 signaling axis, leading to microglial activation and increased expression of proinflammatory cytokines [58]. These findings provide novel insights into the epigenetic regulation of miR-124 and its role in HAND pathogenesis, suggesting potential therapeutic targets for mitigating HIV-1 Tat-mediated neuroinflammation. The implications of these results extend beyond HAND, potentially informing the understanding of other neuroinflammatory conditions and the intricate relationship between microglial activation, epigenetic regulation, and neuronal injury in the context of chronic HIV-1 infection.

Histone acetylation plays a pivotal role in the pathogenesis of HAND, primarily through its influence on gene expression and chromatin structure within the central nervous system (CNS). The dysregulation of histone acetyltransferases (HATs) and histone deacetylases (HDACs) has been implicated in the transcriptional control of HIV and the inflammatory response in the brain, contributing to the neurodegenerative processes observed in HAND. Studies have shown that the HIV Tat protein can interact with HDACs, leading to altered histone acetylation patterns and affecting the expression of neuroinflammatory genes, which are key factors in HAND pathogenesis [59,60]. Furthermore, the use of HDAC inhibitors has been explored as a therapeutic approach to reactivate latent HIV reservoirs in the CNS, highlighting the significance of histone acetylation in both HIV latency and the neuroinflammatory environment conducive to HAND [61].

**Non-coding RNA**: Non-coding RNAs (ncRNAs), particularly microRNAs (miRs) and long non-coding RNAs (lncRNAs), may also play a significant role in the pathogenesis of HAND. These ncRNAs regulate gene expression and have been implicated in the neuroinflammatory processes underlying the disease. A study by Asahchop et al. [62] found nine miRs with increased levels in the HAND group when compared to the non-HAND group. Moreover, lncRNAs, through their interaction with miRs and other molecular targets, contribute to the complex regulatory networks affecting neuronal survival and function during HIV infection [63]. The dysregulation of ncRNAs in HIV-infected individuals can lead to the activation of microglia and astrocytes, promoting neuroinflammation and contributing to the development of HAND [64].

Chronic damage and inflammation exacerbate neurotoxicity risks in HIV patients by disrupting the BBB and contributing to amyloid accumulation [29]. Recent studies have provided further insights into this complex process. For instance, systemic microbial translocation has been associated with increased neuro-inflammation and, occasionally, neuronal injury in HIV infection [65]. The role of systemic lipopolysaccharide (LPS) in contributing to neuro-inflammation remains a subject of investigation. Notably, cerebrospinal fluid (CSF) LPS levels were undetectable in all samples, including those from HIV-infected individuals with dementia. This suggests that while systemic LPS correlates with neuro-inflammation and BBB permeability, it does not directly penetrate the CNS. This underscores the significance of the BBB in mediating the effects of systemic inflammation on the CNS.

Furthermore, the HIV-1 virotoxins, such as gp41, have been shown to induce disorders of the BBB, which is primarily composed of brain microvascular endothelial cells (BMEC) [66]. The alpha7 nicotinic acetylcholine receptor (α7 nAChR) has been identified as an essential regulator of inflammation, contributing to the regulation of NF-κB signaling, neuroinflammation, and BBB disorders caused by microbial (e.g., HIV-1 gp120) and non-microbial factors. Gp120 has been shown to impair the axonal transport of toxic proteins, leading to axonal cytoskeleton breakdown and neuronal loss [67]. Studies on cognitively healthy HIV patients have shown that chronic microglial activation and elevated translocator protein expression are associated with poorer cognitive performance despite effective viral control [68].

Additionally, the concept of a “leaky” BBB facilitating direct viral access to cortical neurons involved in cognitive behaviors has been explored [69]. Viruses may elicit CNS dysfunction either via direct penetration of the BBB or by hijacking the immune cell trafficking network. This highlights the potential for viruses to target the brain as a result of virus-directed natural selection, promoting the longevity and persistence of these pathogens. The development of virus-induced inflammation at the vascular endothelium may enhance normal immune cell trafficking to the brain, a phenomenon reported in cases of Alzheimer’s disease as well.

The infusion of HIV-1 Nef-expressing astrocytes into the rat hippocampus [70] has demonstrated that even a single viral protein can contribute to broader inflammatory responses, increasing BBB permeability and leading to enteropathy and interstitial pneumonitis. This suggests that localized expression of viral proteins like Nef can contribute to the chronic inflammation observed in HIV patients, even those whose viremia is controlled by combination antiretroviral therapy (cART).

Autopsy studies have revealed a predisposition in HIV/AIDS patients to argyrophilic plaque deposition, including extracellular, perivascular, and intraneuronal amyloid plaque deposition [71,72,73]. The severity of HIV-associated neurocognitive disorders correlates with the degree of amyloid deposition, with intracellular amyloid β levels directly associated with age, suggesting that long-term HIV survival may impair protein clearance and exacerbate neuronal damage [27,74,75]. Antiretroviral therapies may influence amyloid plaque deposition, with some therapies impairing amyloid metabolism and potentially increasing the risk of age-related cerebral degenerative changes [76,77]. While earlier hypotheses suggested that reverse transcriptase inhibitors (RTIs), such as Tenofovir, might reduce amyloid deposition by decreasing amyloid precursor protein (APP) gene copies [76], recent studies indicate that RTI therapy may not provide protection against Alzheimer-type amyloidogenesis in the context of HIV infection [78]. This study highlights the complexity of amyloid metabolism in the setting of HIV, suggesting that additional factors beyond RTI therapy may contribute to the development of amyloid plaques in HIV-associated neurocognitive disorders.

Protein homeostasis dysfunction is implicated in the pathophysiology of both HAND and Alzheimer’s disease (AD). HIV regulatory proteins (Tat, Nef, gp120) inhibit proteases that degrade amyloid-beta and disrupt autophagy processes [79,80,81,82], a mechanism similarly observed in AD [83].

## 5. AD Pathogenesis

Pathologically, AD is marked by the accumulation of amyloid-beta (Aβ) plaques and neurofibrillary tangles composed of hyperphosphorylated tau protein in the brain, leading to progressive synaptic damage and neurodegeneration [84,85,86]. The amyloid cascade hypothesis posits that the accumulation of Aβ plaques is the initial event in AD pathogenesis, triggering a series of neurotoxic events, including tau pathology, inflammation, and oxidative stress, ultimately resulting in neuronal death [87]. An extensive review of AD pathogenesis can be found in the literature [88] and is not the focus of this paper.

While no direct causal link between HIV infection and AD has been established, similarities in the pathological processes, especially concerning neuroinflammation, suggest a common mechanism in the progression of both diseases. The fading distinction between HAND and AD is further complicated by studies showing that older PLWH have an increased prevalence of HAND, even after adjusting for infection duration and CD4 count [20,89]. HAND is diagnosed clinically, and current screening tools lack validation. This lack of definitive diagnostic testing for HAND/HAD further confounds the differences between the two disease entities. The differentiation between neurocognitive disorders in PLWH is crucial for preventative management and therapeutic strategies. We hope by delving into the molecular and cellular mechanisms underlying HAND/HAD pathogenesis, and with the availability of large amount of data and review papers in the literature, the scientific community will see the necessity for in-depth investigation of the role of EVs and EV-miRs in ADLD pathogenesis. Indeed, EVs and EV-miRs were mainly investigated for their roles as biomarkers, rather than their pathogenetic effects in AD. Very little, if any, work has been performed to characterize the role of EVs and EV-miRs in ADLD in the setting of HIV infection.

## 6. Biomarkers

Biomarkers represent a critical area of research for distinguishing between AD and HIV-Associated Neurocognitive Disorders (HAND). Core cerebrospinal fluid (CSF) biomarkers, such as total tau (t-tau), hyperphosphorylated tau (p-tau), and beta-amyloid 1–42 (Aβ1-42), have been strongly linked to AD [90,91]. Notably, plasma phosphorylated tau181 (P-tau181) has demonstrated diagnostic accuracy for AD comparable to that of Tau positron emission tomography (Tau PET), a validated diagnostic tool, achieving an area under the curve (AUC) of 0.92–0.96 [92,93]. Previous studies on CSF biomarkers related to amyloid and tau metabolism have observed reductions in amyloid precursor proteins in patients with AIDS dementia complex (ADC) and CNS opportunistic infections, suggesting that CNS immune activation or inflammation may influence neuronal amyloid synthesis [94].

EVs, including exosomes and microvesicles, are integral to cell-to-cell communication and have been implicated as biomarkers for AD and other neurodegenerative diseases [95]. Exosomes, particularly those derived from cerebral spinal fluid (CSF), have been identified to contain specific proteins in higher concentrations in HIV-positive subjects with HAND compared to those without, indicating their utility as diagnostic biomarkers [96,97]. Exosomes play a crucial role in the transport of toxic proteins such as amyloid-beta (Aβ) and tau, which may propagate neurodegenerative changes in AD [98]. The isolation of neuron-derived exosomes (NDEs) using specific antibodies against neural cell adhesion molecules like NCAM or L1CAM has opened new avenues for the study of neurodegenerative diseases [99]. Characterization of these NDEs through techniques such as nanoparticle tracking analysis and Western blots has highlighted their potential as biomarkers for diseases like AD, HAND, and others [100]. Indeed, certain miRs have increased levels in HAND patients [101] and may be used as future biomarkers for the disease. However, the role of EVs and EV-miRs in ADLD, either as biomarkers or as pathogenetic players, has not been elucidated.

In the context of HAND, the study of NDEs has provided insights into the neurocognitive impairment associated with HIV infection. The isolation of NDEs from the plasma of HIV-infected individuals revealed no significant difference in the average diameters of exosomes compared to those from control subjects, although HIV-infected subjects had a higher total number of exosomes. Further characterization showed differences in the levels of tetraspanins and exosomal markers between controls and HIV-infected individuals, suggesting potential biomarkers for HAND [100]. Notably, markers such as amyloid-beta, neurofilament light, and high mobility group box 1 were found to be associated with cognitive impairment; however, they were not specific to HIV infection [102].

Similarly, NDEs have shown promise in the study of Alzheimer’s disease (AD). Recent case–control studies identified NDE biomarkers specific for AD, focusing on pathogenic proteins such as total tau, p181-tau, and Aβ42. These biomarkers were found to be elevated in NDEs from AD patients compared to controls, with abnormalities present even in preclinical stages of AD [98]. Further studies explored other pathways implicated in AD pathogenesis, such as insulin resistance and lysosomal dysfunction, through the measurement of specific mediators in NDEs [103,104]. The use of multiplex proteomics technology allowed for the analysis of a wide range of neurology-related protein biomarkers, revealing a significant enrichment of proteins expressed in neurons or associated with neuronal functions in NDEs. This research underscores the potential of NDEs as biomarkers for early detection and understanding of AD, which may also shed light into its complex pathogenesis.

Differentiating between t-tau and p-tau may aid in distinguishing AD from HAND, as elevated t-tau without concurrent p-tau elevation indicates neural injury and is characteristic of AD. On the other hand, higher levels of t-tau and p-tau have been observed in PLWH with impaired blood–brain barrier (BBB) integrity, which underscore the BBB’s significant role in the pathophysiology of HAND/HAD [105].

Research by Jumare et al. [106] has shown that levels of monocyte activation markers (CD14, CD163) correlate directly with the severity of cognitive impairment in HAND patients. These monocytes, believed to be infected within the bone marrow, can traverse the BBB more easily due to their immature state and reduced exposure to host restriction factors.

Genetic markers may also aid in diagnosis and risk stratification. The apolipoprotein E ε4 allele (APOE4) is the most significant genetic risk factor for sporadic AD, found in 40–65% of all AD patients [107]. It has also been identified as an independent risk factor for HAND in older patients [20,108], associated with poorer cognitive function, diminished white matter integrity, and increased brain atrophy compared to patients without the allele [109]. Furthermore, Soontornniyomkij et al. [108] reported a greater extent of diffuse amyloid plaque deposition in APOE4 carriers. However, studies in the general population have not found a direct association between APOE3 and HAND [110], highlighting the complexity of genetic factors in the pathogenesis of neurocognitive disorders.

## 7. Imaging

Differentiating HAND from AD through imaging techniques is a dynamic field of study. In AD, neuroimaging often reveals distinct patterns, such as hippocampal atrophy in the disease’s early stages, and more extensive cortical atrophy as the disease advances [111,112,113,114,115]. Advanced imaging methods, like positron emission tomography (PET), can detect amyloid-beta deposition, a key feature of AD. Functional imaging techniques, including functional MRI (fMRI) and PET, typically show decreased metabolic activity in the temporal and parietal lobes, indicative of AD [116].

Conversely, imaging findings in HAND tend to be more nuanced and less consistent. Magnetic resonance imaging (MRI) in HAND may reveal diffuse atrophy, which lacks the specificity of the patterns observed in AD. White matter hyperintensities and microstructural changes, identifiable through diffusion tensor imaging (DTI), are common in HAND but are generally not characteristic of early-stage AD [117]. Furthermore, functional imaging in HAND can show distinct brain activation patterns, differing from those seen in AD and reflecting the divergent pathologies of these conditions [118].

Therefore, although HAND and AD both result in cognitive impairment and may share some neuroimaging features, distinct patterns observed in structural and functional imaging can help differentiate between them. It is crucial to consider these imaging results alongside clinical assessments, cognitive evaluations, and other diagnostic standards to distinguish between HAND and AD accurately.

## 8. Conclusions

**Figure 1:**
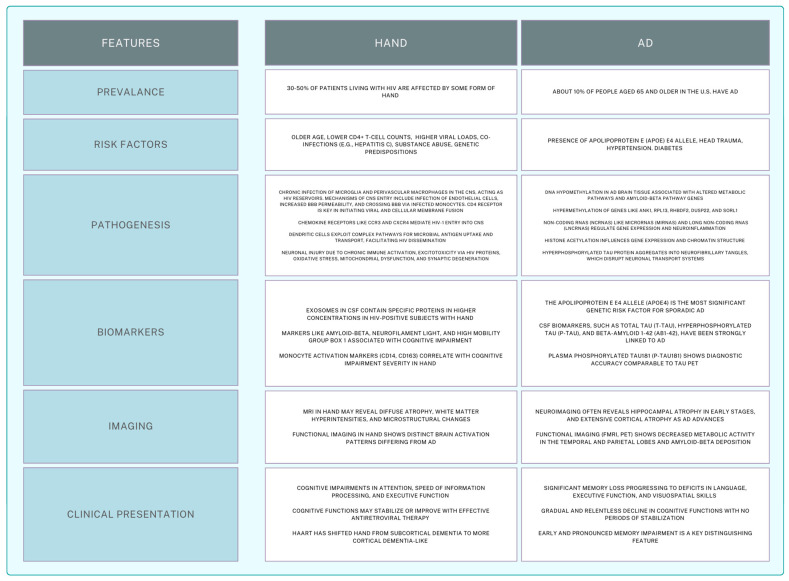
Comparison of features between Alzheimer’s disease and HIV-associated neurocognitive disorders.

**Figure 2:**
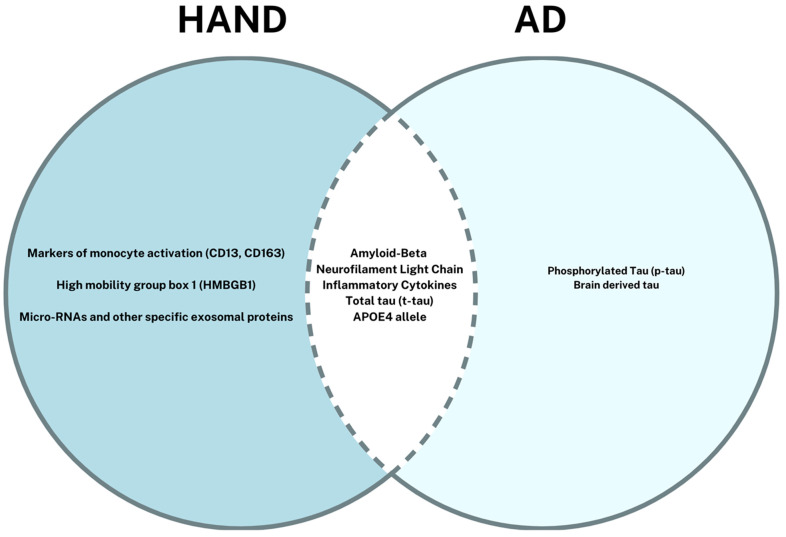
Venn diagram illustrating the similarities and differences in biomarkers HAND and AD.

Differentiating HIV-associated neurocognitive disorder (HAND) from Alzheimer’s disease (AD) remains a critical challenge with significant implications for patient management and therapeutic strategies. This focused review emphasizes the importance of distinguishing between these two neurocognitive conditions, which, despite some overlapping clinical features, exhibit distinct pathophysiological mechanisms and disease progressions. Figure 1 provides a comparison of features between HAND and AD as previously discussed in this review. Figure 2 illustrates the overlap in biomarkers related to each disease process.

HAND is characterized by impairments in attention, information processing speed, and executive function, with cognitive functions potentially stabilizing or improving with effective antiretroviral therapy (ART). Conversely, AD is marked by a gradual and progressive decline in memory and cognitive functions, driven by amyloid-beta deposition and tau pathology. Imaging findings reveal distinct patterns for HAND and AD, which can also aid in their differentiation. Importantly, as PLWH are getting older, ADLD may take the central stage as a main neurological disorder.

It remains unclear why the prevalence of neurocognitive impairment in PLWH remains high, despite effective ART. One potential explanation is the idea of an Alzheimer’s disease-like dementia (ADLD), a disease state that persists beyond active viral infection, signifying that direct viral toxicity may not be the sole cause. While the exact mechanism remains to be elucidated, recent breakthroughs and ongoing research are advancing our understanding of these processes. The evolving theories of HAND neuropathology underscore the complex interplay between HIV proteins, EVs, and CNS cells, offering new insights into the mechanisms of HAND pathogenesis and the potential of exosomes as therapeutic targets and diagnostic tools. It is possible that genetic imprinting, which persists beyond viral clearance and/or chronic inflammation associated with low viral load, can lead to DALD. Emerging research highlights the putative role of EVs in mediating neurocognitive impairment in HIV, even in the absence of active viral infection. EVs facilitate intercellular communication and the transport of pathogenic proteins such as amyloid-beta and tau, as well as HIV-specific factors, contributing to the neuroinflammatory milieu and may serve as both biomarkers and therapeutic targets in ADLD in the setting of HIV infection.

Future research should focus on elucidating the mechanisms by which EVs influence neurodegeneration in HIV, exploring their potential in disease initiation and progression, as well as improving diagnostic accuracy through biomarker analysis. Given the progress of RNA delivery, as evidenced by COVID mRNA vaccines, characterization of the miRs may provide novel therapeutic options for this at-risk population.
